# Immunotherapy and Vaccine Development

**DOI:** 10.1155/2018/8751027

**Published:** 2018-06-13

**Authors:** Carol Leung, Christian Münz, Angelika Riemer

**Affiliations:** ^1^Ludwig Institute for Cancer Research, Nuffield Department of Medicine, University of Oxford, Oxford, UK; ^2^Viral Immunobiology, Institute of Experimental Immunology, University of Zürich, Zürich, Switzerland; ^3^Immunotherapy & Immunoprevention, German Cancer Research Center (DKFZ), Heidelberg, Germany

Recent advances in immunotherapy have led to effective treatments for patients with different diseases including cancer. The use of immune checkpoint-blocking monoclonal antibodies (CPB) and adoptive cellular therapy induces long-term clinical benefits in a number of advanced cancer patients. However, CPB treatment benefits only a small fraction of patients and can be outweighed by autoimmune toxicity, while the adoptive cell therapy approach is complex and requires the culture and transduction of patient-specific immune cells, which can be time-consuming and costly. Cancer vaccines are considered to be a promising alternative, but most trials have failed miserably, apart from the ones targeting virus-associated cancers. A better understanding of the immunological mechanisms is thus crucial in advancing the development of immunotherapy.

In this special issue, seven original research studies and three review articles highlight some recent discoveries in immunotherapy and vaccine development. On vaccine design and development, S. Heinimäki et al. investigated the importance of delivering the VLP-based norovirus vaccine mucosally to induce not only systemic but also the desired mucosal antibody responses. Also, B. Behrouz et al. followed a mucosal vaccination approach and used bivalent flagellin as an immunogen, which induced humoral and cellular immune responses and protected mice from *Pseudomonas aeruginosa*-mediated acute pneumonia. S. Choi et al. assessed if the *Mycobacterium tuberculosis* (Mtb) protein Rv3841, which plays a crucial role in the growth of Mtb, can also serve as a vaccine target. They found that the protein activated dendritic cells (DCs) induced Th1 responses, which could inhibit mycobacterial growth. The topic of antigen production in plants is covered in the article by P.-F. Liu et al. They expressed staphylococcal enterotoxin B in radish leaves by agroinfiltration and showed antibody induction against the antigen after immunizing mice with homogenized leaves. In the area of immunotherapy for autoimmune diseases, J. Yang et al. showed that tolerogenic DCs could expand TGF*β*-induced regulatory T cells (iTregmtDC), which reduced the severity of collagen-induced arthritis in mice. This implies that iTregmtDC might have a therapeutic potential in autoimmune arthritis. For adjuvant studies, J. Laiño et al. described that the dual TLR2/7 ligands, CL413, and CL53, could suppress allergic Th2 immune responses in mice and could act as potential adjuvants for allergy treatment. In the field of animal models for studying immunotherapies and vaccine strategy, C. Leung analyzed the oncofetal antigen ROR1 expression in humanized mice and suggested that humanized mice could be a useful tool to study B cell malignant diseases. In the first of the review articles, A. P. F. Costa et al. carried out a meta-analysis of studies on the safety of the recently introduced nonavalent prophylactic HPV vaccine, which they showed to be as safe as the quadrivalent one. On immunotherapy, S. Sun et al. reviewed the strategies to overcome the toxicities of chimeric antigen receptor (CAR) T cell therapy. In addition, F.-S. Hsu et al. gave a comprehensive review on the use of immune checkpoint inhibitors for treating urothelial carcinoma.

In conclusion, these articles have showcased some novel advances in multiple topics within the field of immunotherapy and vaccine development. We hope the readers will be stimulated by and find applications of the interesting findings.

